# Why Does Doxycycline Pose a Relatively Low Risk for Promotion of *Clostridioides difficile* Infection?

**DOI:** 10.20411/pai.v7i1.512

**Published:** 2022-06-21

**Authors:** Dongyan Xu, Thriveen S.C. Mana, Jennifer L. Cadnum, Abhishek Deshpande, Faezeh Afsari, Naseer Sangwan, Curtis J. Donskey

**Affiliations:** 1 Case Western Reserve University School of Medicine, Cleveland, Ohio; 2 Research Service, Cleveland VA Medical Center, Cleveland, Ohio; 3 Center for Value-Based Care Research, Cleveland Clinic Lerner College of Medicine, Cleveland, Ohio; 4 Lerner Research Institute/Lerner College of Medicine of Case Western Reserve University, Cleveland, Ohio; 5 Geriatric Research, Education and Clinical Center, Cleveland VA Medical Center, Cleveland, Ohio

**Keywords:** *Clostridioides difficile*, doxycycline, azithromycin, microbiome

## Abstract

**Background:**

Clinical studies suggest that doxycycline poses a low risk for promotion of *Clostridioides difficile* infection, but the microbiologic explanation for this finding is unclear.

**Methods:**

Mice treated with oral doxycycline, oral azithromycin, subcutaneous ceftriaxone, doxycycline plus ceftriaxone, or azithromycin plus ceftriaxone were challenged with 10^4^ colony-forming units of 2 different *C. difficile* strains on day 2 of 5 of treatment. The concentration of *C. difficile* was measured in stool 2 and 5 days after challenge. The impact of the treatments on the microbiota was assessed by sequencing.

**Results:**

Doxycycline and azithromycin treatment did not promote colonization by either *C. difficile* strain in comparison to saline controls. Doxycycline treatment significantly reduced ceftriaxone-induced overgrowth of a *C. difficile* strain with doxycycline minimum-inhibitory concentration (MIC) of 0.06 µg/mL (*P*<0.01) but not a strain with doxycycline MIC of 48 µg/mL (*P*>0.05); azithromycin treatment did not reduce ceftriaxone-induced overgrowth of either strain. 16S rRNA amplicon sequencing revealed significantly lower bacterial diversity in the stool of ceftriaxone-treated mice, in comparison to doxycycline-treated and azithromycin-treated mice.

**Conclusions:**

These findings suggest that doxycycline may have a low propensity to promote *C. difficile* colonization because it causes relatively limited alteration of the indigenous microbiota that provide colonization resistance and because it provides inhibitory activity against some *C. difficile* strains.

## INTRODUCTION

*Clostridioides difficile* is the most important cause of healthcare-associated infectious diarrhea in developed countries [1]. Antibiotics that disrupt the indigenous intestinal microbiota are the major cause of *C. difficile* infection (CDI) [2–4]. Although all classes of antibiotics have been associated with CDI, cephalosporins, fluoroquinolones, clindamycin, and penicillins are generally considered the agents that pose the greatest risk [2–4]. To develop effective antimicrobial steward-ship interventions for CDI, there is a need to identify antibiotics that have a relatively low propensity to disrupt the intestinal microbiota and cause CDI.

Doxycycline is an oral antibiotic used for treatment of a variety of conditions including community-acquired pneumonia, bronchitis, and soft tissue infections [2]. Several studies have reported that doxycycline may pose a relatively low risk for CDI [2, 5–8], but the microbiological explanation for this finding is unclear. One potential explanation is that doxycycline causes relatively little disruption of the indigenous intestinal microbiota [9, 10]. A second possibility is that doxycycline has activity against many *C. difficile* strains and might inhibit colonization if exposure occurs during therapy when the drug is present in the intestinal tract [3, 4, 6, 11, 12]. In the current study, we used an established mouse model to test the hypothesis that doxycycline has a relatively low risk for promotion of *C. difficile* colonization due to both limited disruption of the intestinal microbiota and inhibitory activity against *C. difficile*.

## METHODS

### The pathogens studied

VA17 is a clinical epidemic North American pulsed-field gel electrophoresis type 1 (NAP1) *C. difficile* strain. And 386 is a restriction endonuclease analysis B2 toxin A and B positive *C. difficile* isolate with tetracycline minimum-inhibitory concentration (MIC) of 48 by Etest. *C. difficile* spores were prepared as previously described [13].

### Susceptibility testing and bioassay for antibiotic concentrations

Broth dilution MICs of the test antibiotics for the test organisms were determined using standard methods for susceptibility testing of aerobic and anaerobic bacteria [14]. The concentrations of doxycycline and azithromycin in stool were determined by an agar diffusion assay with *Bacillus subtilis* as the indicator strain [14].

### Quantification of C. difficile in stool

Fresh stool specimens were processed as described elsewhere [11, 12]. To quantify *C. difficile*, diluted samples were plated onto pre-reduced cycloserine-cefoxitin-brucella agar containing 0.1% taurocholic acid and 5 mg/mL lysozyme (*C. difficile* brucella agar), respectively. The plates were incubated for 48 hours, and the number of colony-forming units (CFU) of *C. difficile* per gram of sample was calculated.

### Antibiotic dose selection

The Animal Care Committee of the Cleveland Veterans Affairs Medical Center approved the experimental protocol. Female CF-1 mice (5 per group) weighing ~30 g (Harlan Sprague-Dawley, Indianapolis, IN) were housed in individual cages. Dose finding experiments were run to determine the amount of doxycycline to be dosed to result in stool concentrations in mice similar to those measured in humans (ie, mean 3.1 mg in stool on day 1 of treatment with 100 mg per day to human volunteers) [9]. Mice (5 per group) received a single oral administration of doxycycline in a dose equivalent to the usual human dose on a mg per kg basis (0.1 mg) or 5X (0.5 mg), 12X (1.2 mg), and 20X (2 mg) the usual human dose on a mg per kg basis. Fecal pellets were collected at 4, 8, and 24 hours after dosing. Fecal levels of doxycycline were measured by bioassay as described previously.

For azithromycin, we used a dose equivalent to the dose selected for doxycycline (5X the usual human dose on a mg per kg basis). For ceftriaxone, a dose equivalent to the usual human dose on a mg per kg basis was used because this dose has been used in previous mouse model studies and results in alteration of the microbiota of mice that is similar to alteration in ceftriaxone-treated humans [15].

### Effect of the antibiotics on establishment of colonization by C. difficile

Mice (5 per group) received 5 days of daily treatment with oral PBS (0.1 mL), doxycycline (0.5 mg in 0.1 mL PBS), oral azithromycin (1.25 mg in 0.1 mL PBS), subcutaneous ceftriaxone (1 mg in 0.1 mL PBS), subcutaneous ceftriaxone (1 mg) plus oral doxycycline (0.5 mg), or subcutaneous ceftriaxone (1 mg) plus oral azithromycin (1.25 mg). On day 2 of antibiotic treatment (4 to 6 hours after the antibiotic dose), mice received 10,000 CFU of *C. difficile* 368 or VA17 spores by orogastric gavage. The concentration of *C. difficile* in stool was measured 2 and 5 days after gavage of the spores. The rationale for administering azithromycin and doxycycline in combination with ceftriaxone was to determine if these agents have sufficient inhibitory activity to prevent ceftriaxone-induced overgrowth of *C. difficile*. For the VA17 strain, additional groups of mice were challenged with the VA17 strain during treatment with subcutaneous ceftriaxone plus 20X the usual human dose of doxycycline or azithromycin (ie, oral doxycycline 2 mg or oral azithromycin 5 mg).

### Effect of antibiotic treatment on the intestinal microbiota

Mice received daily dosing for 2 days with PBS (0.1 mL), doxycycline (0.5 mg or 5X the usual human dose in 0.1 mL PBS) by orogastric gavage, azithromycin (1.25 mg or 5X the usual human dose in 0.1 mL PBS) by orogastric gavage, or ceftriaxone (1 mg or 1X the usual human dose in 0.1 mL PBS) subcutaneously. Stool samples (~100 mg total) were collected 4 to 6 hours after the second daily dose for sequencing analysis.

### DNA Extraction and 16S rRNA amplicon sequencing

DNA Extraction and 16S sRNA amplicon sequencing were performed as described previously [16]. Briefly, DNA was isolated from stool samples using the QIAamp DNA Microbiome kit (Qiagen). The isolated microbial gDNA was checked for signs of degradation and quantified using the Bio-analyzer (Agilent) to ensure accurate sample input for the initial PCR step. A nested PCR method was used for amplification of the V4 region of the 16S rRNA gene and the addition of Illumina Nextera Unique Dual indexes. Afterwards, each library underwent standard quality control procedures checking for sample concentration and sample quality. Each library was pooled together ensuring equal sample distribution amongst sequencing reads. Amplicon sequencing was performed on an Illumina MiSeq with a 2x150 read length.

### Data analysis

One-way analysis of variance (ANOVA) was performed to compare concentrations of organisms among the treatment groups. *P*-values were adjusted for multiple comparisons using the Scheffe correction. Computations were performed with the use of Stata (version 5.0, Stata, College Station, Texas).

For analysis of the sequencing data, individual fastq files without non-biological nucleotides were processed using the Divisive Amplicon Denoising Algorithm (DADA) pipeline [17]. The output of the dada2 pipeline (feature table of amplicon sequence variants [an ASV table]) was processed for alpha and beta diversity analysis using *phyloseq* [18] and microbiomeSeq (http://www.github.com/umerijaz/microbiomeSeq) packages in R. Alpha diversity estimates were measured within group categories using estimate richness function of the *phyloseq* package. Canonical correspondence analysis (CCA) was performed using Bray-Curtis dissimilarity matrix between groups and visualized by using *ggplot2* package [19]. Differential abundance analysis was performed using ANOVA in R software (The R Foundation for Statistical Computing, Vienna, Austria). As appropriate, we adjusted for multiple comparisons using the BH FDR method while performing multiple testing on taxa abundance across groups [20]. Permutational multivariate analysis of variance (PERMANOVA) was performed on all coordinates obtained during CCA.

## RESULTS

### Susceptibility testing

For *C. difficile* VA17, the MICs of doxycycline, azithromycin, and ceftriaxone were 0.06, 16, and >64 µg/mL, respectively. For *C. difficile* 368, the MICs of doxycycline, azithromycin, and ceftriaxone were 48, 500, and >64 µg/mL, respectively.

### Doxycycline dose selection

No doxycycline was detected in the stool when doxycycline was administered as a single dose equivalent to the usual daily human dose. At 5X the usual daily human dose, doxycycline was detected at mean concentrations of 15.9 and 11.4 µg per mg stool at 4 and 8 hours post-dosing, respectively; no doxycycline was detected in samples collected 24 and 48 hours post-dosing. At 12X the usual human dose, doxycycline was detected at mean concentrations of 10.7, 26.3, and 5.1 µg per mg stool at 4, 8, and 24 hours post-dosing, respectively. At 20X the usual human dose, doxycycline was detected at mean concentrations of 14.6, 29.9, and 14.9 µg per mg stool at 4, 8, and 24 hours post-dosing, respectively. Based on these results and prior stool concentrations in humans, the 5X dose was chosen for subsequent testing.

### Effect of antibiotic treatment on establishment of colonization by *C. difficile*

**Figure 1.**
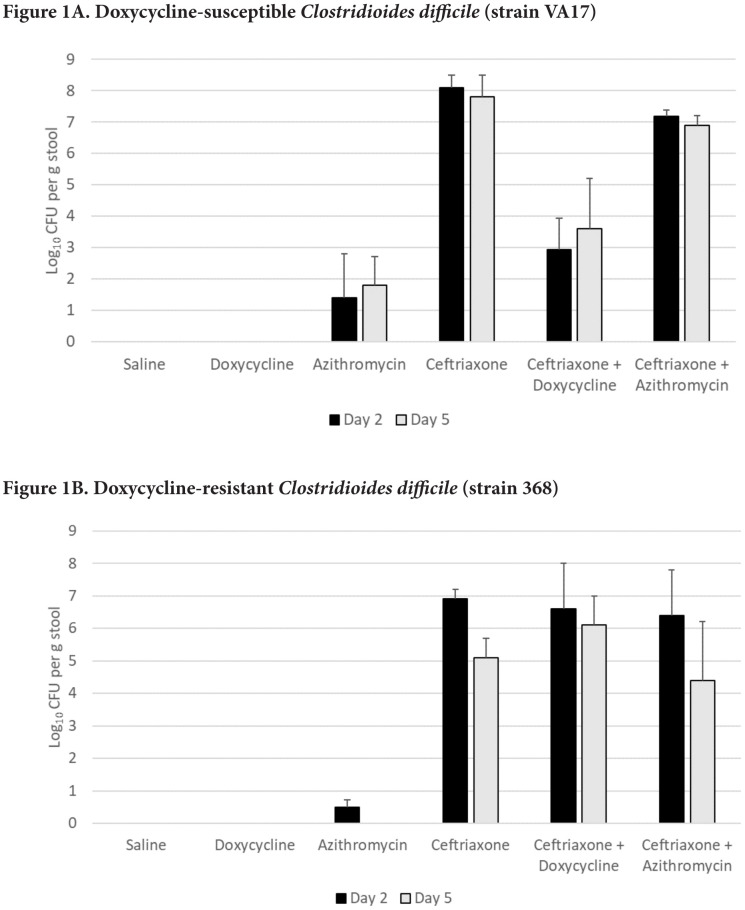
Effect of antibiotic treatment on establishment of colonization by *Clostridioides difficile*. Mice (5 per group) received 10,000 colony-forming units (CFU) of *C. difficile* VA17 (A) or 368 (B) on day 2 of 5 of daily antibiotic treatment. The concentration of *C. difficile* in stool was measured 2 days after gavage of the *C. difficile* test strain. Error bars represent standard error.

Figure 1A shows the impact of antibiotic treatment on establishment of *C. difficile* VA17 after exposure on day 2 of 5 of treatment. Ceftriaxone, ceftriaxone plus doxycycline, and ceftriaxone plus azithromycin promoted overgrowth of *C. difficile* VA17 in comparison to saline controls (*P*<0.05), whereas azithromycin (*P*=0.32) and doxycycline (*P*>.99) did not. In comparison to ceftriaxone-treated mice, ceftriaxone plus doxycycline-treated mice significantly reduced *C. difficile* concentrations (*P*<0.01), whereas ceftriaxone plus azithromycin-treated mice did not (*P*=0.09). When the dose of azithromycin and doxycycline was increased to 20-times the usual human dose on a mg per kg basis, ceftriaxone plus doxycycline-treated mice had no detectable *C. difficile* VA17 colonization, but all ceftriaxone plus azithromycin-treated mice had high-level colonization (>6.3 log_10_ CFU per g stool) (data not shown).

Figure 1B shows the impact of antibiotic treatment on establishment of *C. difficile* 368 after exposure on day 2 of 5 of treatment. Ceftriaxone, ceftriaxone plus doxycycline, and ceftriaxone plus azithromycin promoted overgrowth of *C. difficile* 368 in comparison to saline controls (*P*<0.05), whereas azithromycin and doxycycline did not (*P*>0.05). In comparison to ceftriaxone-treated mice, ceftriaxone plus doxycycline-treated mice and ceftriaxone plus azithromycin-treated mice had similar *C. difficile* concentrations (*P*>0.05).

### Sequencing analysis of the stool microbiota

**Figure 2.**
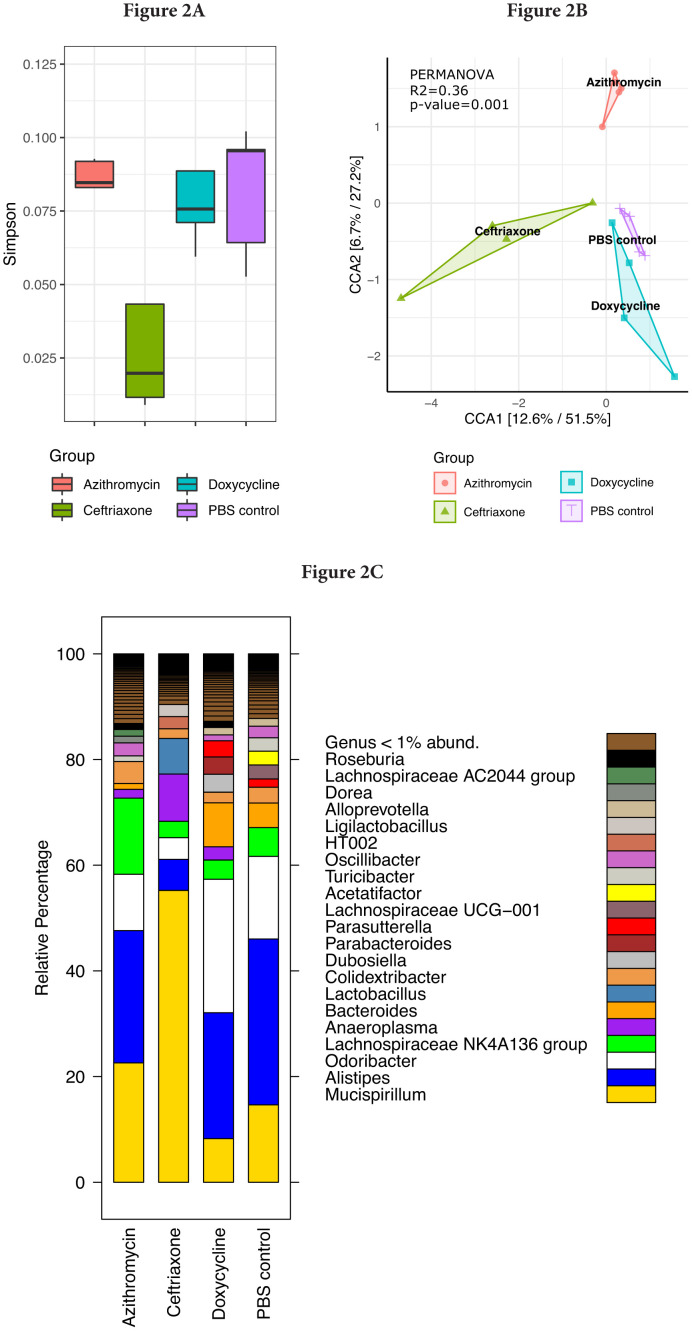
Impact of treatment with phosphate-buffered saline (PBS), doxycycline, azithromycin, or ceftriaxone on the total bacterial diversity in the stool of mice. A) Alpha Simpson diversity index; B) Beta diversity canonical correspondence analysis (CCA); C) Relative percentage abundance of different bacterial taxa. Black horizontal lines associated with the box plots represent median values.

Figure 2 shows the impact of treatment on the total bacterial diversity in the stool of mice treated with normal saline, doxycycline, azithromycin, or ceftriaxone. Alpha (Simpson diversity index) and beta diversity (canonical correspondence analysis [CCA]) analysis of 16s rRNA gene amplicon sequencing data revealed differential community shift patterns in azithromycin, ceftriaxone, doxycycline, and PBS control samples. Doxycycline and azithromycin samples showed alpha diversity patterns similar to the PBS controls, suggesting that both antibiotics caused relatively limited disruption of the microbiota. In contrast, ceftriaxone treatment caused a marked (Wilcoxon Rank sum test, *P*<0.05) decrease in the alpha diversity as compared to doxycycline and azithromycin treatment. For ceftriaxone-treated mice, the relative percentage of Mucispirillum was substantially greater than in the other treatment groups, whereas the relative percentage of Alistipes was substantially reduced (Figure 2C).

**Figure 3.**
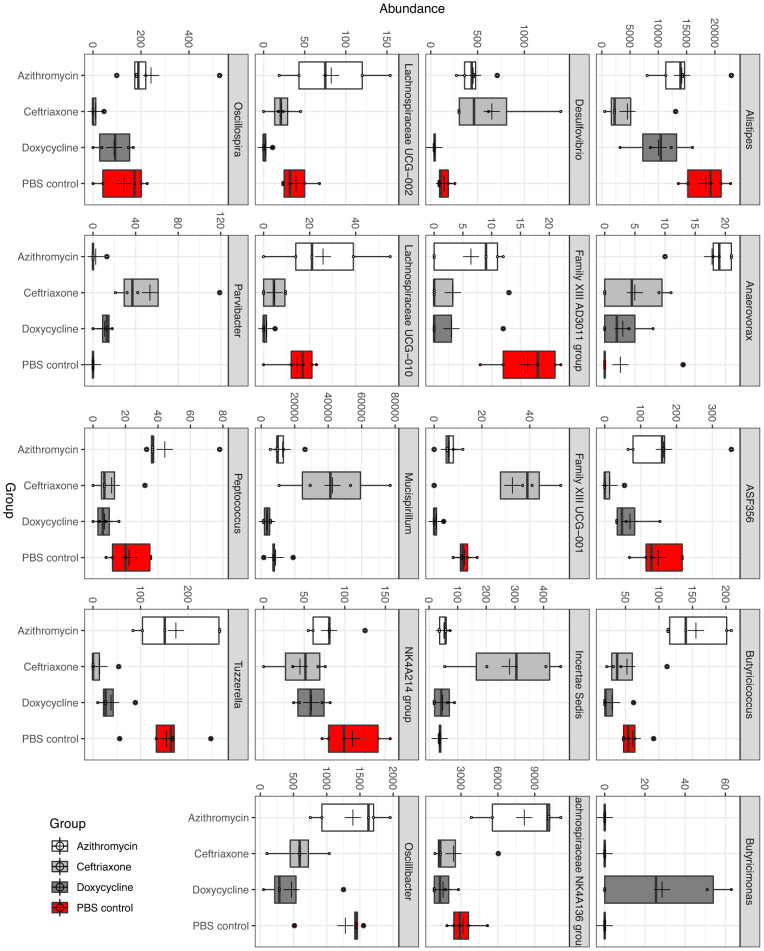
Differential abundance analysis (*P*<0.05, ANOVA, Benjamini-Hochberg (BH)) highlighting 19 taxa at genus level whose abundance was differentially altered in the antibiotic treatment groups versus the phosphate-buffered saline (PBS) control group.

Differential abundance analysis (*P*<0.05, ANOVA, Benjamini-Hochberg (BH]) highlighted 19 taxa at genus level whose abundance was differentially altered in the antibiotic treatment groups versus the PBS control group (Figure 3). In comparison to the PBS controls, ceftriaxone-treated mice had significantly increased abundance of Mucispirillum, Family XIII UCG-001, and Parvibacter, whereas the abundance of Alistipes, ASF 358, Family XIII AD3011, NK 4A214 group, Oscillibacter, and Tuzzerella was significantly decreased. In comparison to the PBS controls, doxycycline-treated mice had significantly reduced abundance of Family XIII UCG-001, Family XIII AD3011, Lachnospiraceae UCG-002, and Tuzzerella. In comparison to the PBS controls, azithromycin-treated mice had significantly increased abundance of Anaerovorax, Butyricicoccus, and Lachnospiraceae NK4A136 group, whereas the abundance of Family XIII AD3011 was significantly decreased.

## DISCUSSION

In a mouse model, treatment with oral doxycycline did not promote colonization by doxycycline-susceptible or doxycycline-resistant strains of *C. difficile*. Doxycycline administered in combination with ceftriaxone reduced ceftriaxone-induced overgrowth of the susceptible but not the resistant strain of *C. difficile*, demonstrating that doxycycline achieved sufficient concentrations in the intestinal tract to reduce colonization by the doxycycline-susceptible *C. difficile* strain. These results suggest that doxycycline may have a low propensity to promote *C. difficile* both because it causes relatively little alteration of the indigenous microbiota that provide colonization resistance against *C. difficile*, and because it provides inhibitory activity against some strains of *C. difficile*.

Our findings are consistent with previous evidence that doxycycline treatment can alter the intestinal microbiota, but to a relatively modest degree in comparison to antibiotics such as ceftriaxone [9, 10, 21–23]. Although emergence of doxycycline-resistant microorganisms was a common finding in studies of healthy volunteers, only relatively minor alterations in the concentration of different components of the microbiota were reported based on culture results [9, 10, 21]. Doxycycline treatment also only minimally altered short-chain fatty acid and bile pigment excretion [21, 22].

In recent surveillance studies, a majority of *C. difficile* isolates have been susceptible to tetracyclines [4, 6, 7]. In a recent systematic review and meta-analysis of antimicrobial resistance in *C. difficile*, tetracycline had a calculated weighted pooled resistance of 20% [7]. These findings suggest that doxycycline may have sufficient activity to inhibit many currently circulating strains of *C. difficile*. Macrolide antibiotics also have variable activity against *C. difficile* [4], and therefore we cannot exclude the possibility that azithromycin might inhibit some circulating *C. difficile* strains.

Given the evidence that doxycycline may have a low propensity to promote CDI, it has been proposed that doxycycline might be preferred over azithromycin for treatment of community-acquired pneumonia [6, 7, 24]. In clinical studies, macrolides have been associated with an increased risk for CDI in comparison to tetracyclines [2, 5–8]. In the current study, neither drug promoted significant overgrowth of *C. difficile*, although low levels of *C. difficile* were detected in the stool of azithromycin-treated but not doxycycline-treated mice. Additional studies are needed to determine if doxycycline has a relatively low propensity to promote intestinal colonization with other healthcare-associated pathogens. In mice, doxycycline did not promote colonization by vancomycin-resistant enterococci (VRE) or *Klebsiella pneumoniae*, while azithromycin promoted VRE but not *K. pneumoniae* (author's unpublished data).

One notable finding from the sequencing analysis was that ceftriaxone treatment resulted in a significant increase in the proportion of *Mucispirillum* spp. *Mucispirillum schaedleri* (phylum Deferribacteres) is present in the intestinal microbiota of mice and other animals and is a low-abundance member of the human intestinal microbiota associated with the intestinal mucosa [25, 26]. *M. schaedleri* has been shown to protect mice against enteric *Salmonella enterica* serovar Typhimurium infection by interfering with pathogen invasion and virulence factor expression [25]. Additional studies are needed to determine if *M. schaedleri* plays a role in prevention of colonization by healthcare-associated pathogens such as *C. difficile*.

Our study has some limitations. We studied only 2 strains of *C. difficile*. The study was conducted using a mouse model with healthy mice dosed once daily with the antibiotics. Although levels of doxycycline in stool samples were similar to levels previously reported in humans [9, 10, 23], antibiotic excretion in the intestinal tract of mice and humans may differ. Therefore, additional studies will be required to confirm that the findings are applicable to patients. Finally, the challenge with pathogens occurred during antibiotic treatment. Antibiotic-induced disruption of the microbiota may result in a vulnerable period for establishment of colonization after completion of antibiotic treatment [27]. Further studies are needed to assess establishment of colonization when pathogen challenge occurs after completion of treatment.
